# Acquired reactive perforating collagenosis treated with nemolizumab

**DOI:** 10.1016/j.jdcr.2025.12.044

**Published:** 2026-01-05

**Authors:** Shehani T. Perera, Sofia Vescovacci, Sara Kaye Givens, Marc Inglese, András Schaffer, Austinn Miller

**Affiliations:** a Florida State University College of Medicine, Tallahassee, Florida; b Dermatology Associates of Tallahassee, Tallahassee, Florida; c Department of Dermatology, University of Central Florida HCA Florida Healthcare Graduate Medical Education, Tallahassee, Florida

**Keywords:** acquired reactive perforating collagenosis, end-stage renal disease, IL-31, nemolizumab, perforating disorders

## Introduction

Perforating dermatoses represent a heterogeneous group of skin diseases characterized by transepidermal elimination of dermal components such as collagen, elastic fibers, or keratin. Clinically, they present with intensely pruritic, umbilicated papules or nodules with keratotic plugs.^1^

Acquired reactive perforating collagenosis (ARPC) is distinguished from other perforating disorders by its selective elimination of collagen bundles. It predominantly occurs in adults with diabetes mellitus and chronic kidney disease and is perpetuated by the itch-scratch cycle.^1^, ^2^, ^3^ ARPC may share clinical and pathogenic features with prurigo nodularis (PN) and has been proposed as an umbilicated variant within the PN spectrum.^3^

Conventional therapies including topical corticosteroids, phototherapy, and antihistamines often yield incomplete control.^1^^,^^2^ Nemolizumab, a monoclonal antibody targeting interleukin 31 (IL-31) receptor A, reduces chronic itch and has shown improvement in dialysis-associated pruritus and ARPC.^2^, ^3^, ^4^ We present a dialysis-dependent patient with clinicopathologically confirmed ARPC with PN overlap, unresponsive to topical corticosteroids, who improved rapidly after nemolizumab initiation.

## Case presentation

**Fig 1:**
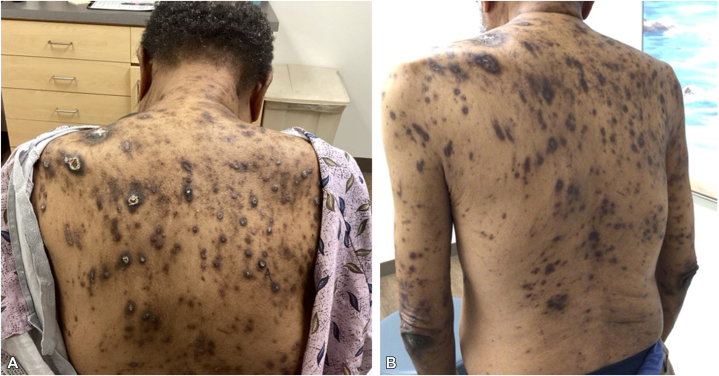
**A,** Initial presentation demonstrating numerous nodules consistent with acquired reactive perforating collagenosis and prurigo nodularis. **B,** Two months postnemolizumab initiation showing marked clinical improvement with residual hyperpigmentation.

A 68-year-old Black man with diabetes mellitus and end-stage renal disease (ESRD) on hemodialysis presented with 7 months of severe generalized pruritus unresponsive to high-potency topical corticosteroids. No oral antipruritic, phototherapy, or systemic agents were given because of comorbidities and patient preference. Examination revealed over 100 discrete hyperpigmented papules and nodules on the trunk and extremities (Fig 1). Many were umbilicated with central keratotic plugs and crusts.

**Fig 2:**
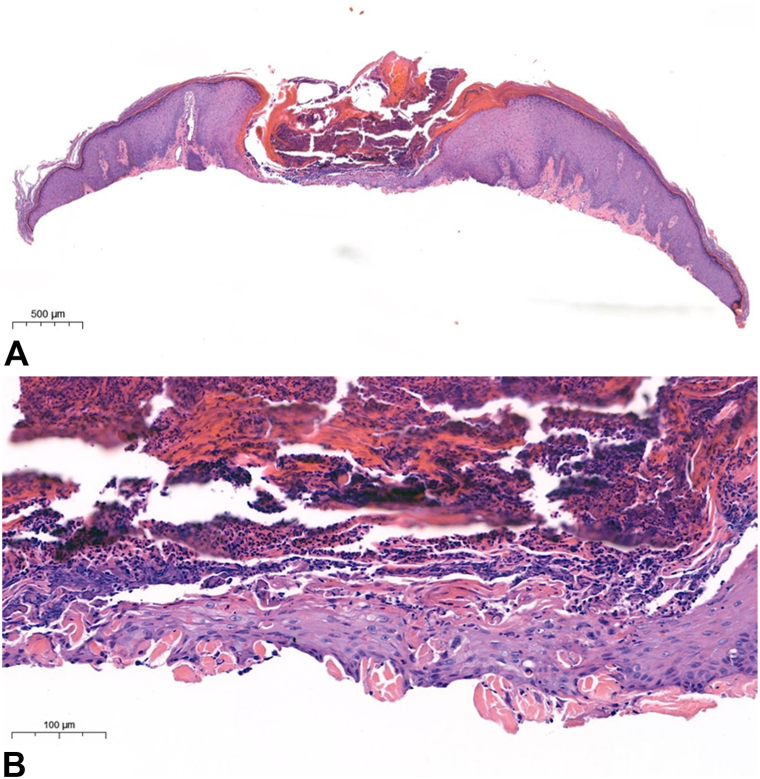
**A,** Hyperkeratosis overlying cup-shaped central depression with basophilic material and surrounding epidermal hyperplasia. **B,** Transepidermal elimination of collagen fibers. (**A** and **B,** Hematoxylin-eosin stain; original magnifications: **A,** '3; **B,** '24.)

Biopsy of an umbilicated lesion demonstrated ulceration with inflammatory crust and degenerating collagen undergoing transepidermal elimination (Fig 2). These findings are diagnostic of ARPC.^3^^,^^5^ However, a biopsy from an early, nonumbilicated papule showed features more consistent with PN without transepidermal elimination. Given most lesions demonstrated umbilication with central keratotic plugs, the overall presentation was most consistent with ARPC with PN overlap.

Nemolizumab was initiated with a 60-mg loading dose subcutaneously followed by 30 mg injections every 4 weeks.^4^

At 1 month, the patient reported marked reduction in pruritus and lesion count. By 2 months, dramatic clinical improvement was noted, with only residual hyperpigmentation. No adverse reactions were observed.

## Discussion

ARPC frequently occurs in patients with diabetes mellitus and ESRD, with increased prevalence among dialysis cohorts.^1^^,^^2^ Clinically and histologically, ARPC and PN share features of chronic itch and trauma-induced transepidermal elimination.^3^ Emerging evidence suggests that ARPC may represent a subtype or variant within the PN spectrum, sometimes termed the “umbilicated type of prurigo.”^1^, ^2^, ^3^ Both conditions are unified by their pathomechanism of chronic pruritus and repetitive scratching. This process induces transepidermal elimination of dermal components—collagen in ARPC and necrotic keratinocytes in PN.^2^, ^3^, ^4^ Systemic diseases such as diabetes mellitus and ESRD further perpetuate this cycle by impairing wound repair and promoting metabolic dysregulation.

IL-31 acts as a key mediator in chronic itch, with elevated serum levels in dialysis patients correlating with pruritic severity.^6^^,^^7^ IL-31 expression also correlates with *μ*-opioid receptor dysregulation in uremic pruritus, linking neuronal itch signaling with cytokine-driven inflammation. Nemolizumab targets the IL-31 receptor and alleviates itch and inflammation, disrupting the itch-scratch cycle.^5^

Traditional ARPC therapies are often unsafe in ESRD because of impaired drug clearance and increased risk of adverse effects.^1^^,^^2^ Biologic therapies such as dupilumab have shown benefit, supporting T helper 2 pathway involvement.^5^ Nemolizumab offers a kidney-safe option, as monoclonal antibodies are not renally cleared or removed by dialysis.^3^^,^^7^

Although immunohistochemical staining was not performed in this case, studies demonstrate reduction of transepidermal collagen elimination and lesion resolution after IL-31 blockade, suggesting nemolizumab may directly affect ARPC pathogenesis beyond itch control.^5^^,^^7^

Although improvement in this case may reflect modulation of PN or a direct effect on ARPC, prior reports describe morphologic resolution of ARPC lesions with nemolizumab, supporting a broader role for IL-31 blockade in perforating dermatoses.^5^^,^^7^ Regular follow-up is warranted, as recurrence often parallels systemic disease activity.^1^^,^^2^

## Conclusion

Nemolizumab resulted in rapid pruritic relief and morphologic clearing of ARPC lesions in a dialysis-dependent patient with ARPC-PN overlap refractory to topical therapy.^4^^,^^5^ These findings support IL-31 blockade as a rational, kidney-safe therapeutic approach for perforating dermatoses associated with systemic disease.^6^^,^^7^

## Conflicts of interest

None disclosed.

## References

[1] Kawakami T., Akiyama M., Ishida-Yamamoto A. (2020). Clinical practice guide for the treatment of perforating dermatosis. J Dermatol.

[2] Kestner R.I., Ständer S., Osada N., Ziegler D., Metze D. (2017). Acquired reactive perforating dermatosis is a variant of prurigo nodularis. Acta Derm Venereol.

[3] Ohmori S., Sawada Y. (2023). Perforating dermatosis in a patient on haemodialysis successfully treated with nemolizumab. Clin Exp Dermatol.

[4] Yamada K., Baba A., Kanekura T. (2024). A giant variant of acquired reactive perforating collagenosis successfully treated with nemolizumab. J Eur Acad Dermatol Venereol.

[5] Gil-Lianes J., Loughlin C.R.M., Mascaró J.M. (2022). Reactive perforating collagenosis successfully treated with dupilumab. Australas J Dermatol.

[6] Świerczyńska K., Krajewski P.K., Nowicka-Suszko D., Białynicki-Birula R., Krajewska M., Szepietowski J.C. (2022). The serum level of IL-31 in patients with chronic kidney disease-associated pruritus: what can we expect?. Toxins (Basel).

[7] Kinugasa E, Igawa K, Shimada H (2021). Anti-pruritic effect of nemolizumab in hemodialysis patients with uremic pruritus: a phase II, randomized, double-blind, placebo-controlled clinical study. Clin Exp Nephrol.

